# A panel of DNA methylation markers for the classification of consensus molecular subtypes 2 and 3 in patients with colorectal cancer

**DOI:** 10.1002/1878-0261.13098

**Published:** 2021-09-30

**Authors:** Inge van den Berg, Marcel Smid, Robert R. J. Coebergh van den Braak, Mark A. van de Wiel, Carolien H. M. van Deurzen, Vanja de Weerd, John W. M. Martens, Jan N. M. IJzermans, Saskia M. Wilting

**Affiliations:** ^1^ Department of Surgery Erasmus MC ‐ University Medical Center Rotterdam The Netherlands; ^2^ Department of Medical Oncology Erasmus MC Cancer Institute University Medical Center Rotterdam The Netherlands; ^3^ Department of Epidemiology & Data Science Amsterdam University Medical Center Amsterdam Public Health research institute The Netherlands; ^4^ Department of Pathology Erasmus MC – University Medical Center Rotterdam The Netherlands

**Keywords:** colon cancer, consensus molecular subtypes, marker panel, methylation

## Abstract

Consensus molecular subtypes (CMSs) can guide precision treatment of colorectal cancer (CRC). We aim to identify methylation markers to distinguish between CMS2 and CMS3 in patients with CRC, for which an easy test is currently lacking. To this aim, fresh‐frozen tumor tissue of 239 patients with stage I‐III CRC was analyzed. Methylation profiles were obtained using the Infinium HumanMethylation450 BeadChip. We performed adaptive group‐regularized logistic ridge regression with post hoc group‐weighted elastic net marker selection to build prediction models for classification of CMS2 and CMS3. The Cancer Genome Atlas (TCGA) data were used for validation. Group regularization of the probes was done based on their location either relative to a CpG island or relative to a gene present in the CMS classifier, resulting in two different prediction models and subsequently different marker panels. For both panels, even when using only five markers, accuracies were > 90% in our cohort and in the TCGA validation set. Our methylation marker panel accurately distinguishes between CMS2 and CMS3. This enables development of a targeted assay to provide a robust and clinically relevant classification tool for CRC patients.

## Graphical abstract

Consensus molecular subtypes (CMSs) can guide precision treatment of colorectal cancer (CRC). However, an easy test to distinguish between CMS2 and CMS3 in patients with colorectal cancer is currently lacking. We developed two methylation marker panels distinguishing between CMS2 and CMS3, both with accuracies > 90% in our cohort and in TCGA validation set. This enables development of a targeted assay to provide a robust and clinically relevant classification tool for CRC patients.

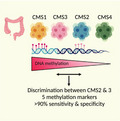

**Abbreviations**
5’UTR: 5’‐untranslated region
AUC: area under ROC curve
BMIQ: beta‐mixture quantile
BP: base pair
CGI: CpG island
CIMP: CpG island methylator phenotype
CMS: consensus molecular subtype
CRC: colorectal cancer
FDR: false discovery rate
grridge: group‐regularized ridge regression analysis
MCL: Markov cluster algorithm
N‐shelf: northern shelf
N‐shore: northern shore
PCA: principal component analysis
RF: random forest classifier
ROC curve: receiver operating characteristic curve
SCNAs: somatic copy‐number alterations
S‐shelf: southern shelf
S‐shore: southern shore
SSP: single‐sample predictor
TCGA: The Cancer Genome Atlas
TSS: transcription start site

## 1 Introduction

The consensus molecular subtype (CMS) classification is currently considered to be the most robust molecular stratification in colorectal cancer (CRC) with significant differences in prognosis [1]. Besides the prognostic value, literature provides some support for a predictive value of CMS in response to systemic treatment [2]. The FOxTROT study (NCT00647530) and currently ongoing CONNECTION‐II trial (NTR NL8177) are expected to determine the true predictive value of CMS in response to chemotherapy. However, in general practical and affordable tests to determine CMS will greatly aid in establishing the clinical value of these molecular subtypes as these will enable routine determination of CMS in ongoing CRC research. The gold‐standard classification strategy relies on genome‐wide RNA expression data from sufficient quantities of fresh‐frozen bulk tumor, which hampers widespread implementation. In addition, different methods can be used to classify CMS on RNA data, which inherently causes differences in CMS calling per method. These classification methods include a Markov cluster algorithm (MCL), which is the algorithm applied by Guinney *et al*., a random forest classifier (RF, based on MCL calls), and a classifier by similarity to centroid approach (single‐sample predictor, SSP) which calls each sample independent from other samples. An affordable, robust, and practical classification assay is needed to enable both retrospective and prospective investigations of the predictive value of the CMS classification and advance its use in clinical practice. For CMS1, MSI can be used as a surrogate marker given the high incidence of MSI in CMS1 tumors and the low incidence of MSI in CMS2‐4 [3]. Sufficient evidence from both observational studies and randomized clinical trials is available to justify that MSI tumors represent a separate entity requiring a different treatment strategy, irrespective of their CMS classification [4, 5]. MSI testing can be done very robustly and is included in the international clinical guidelines [6]. For CMS4, an immunohistochemistry‐based classifier and an RT‐qPCR test have been described and validated [7, 8]. However, a more practical test to distinguish between CMS2 and CMS3 remains to be identified. Given the low specificity of the original CMS classification algorithm on archival formalin‐fixed paraffin‐embedded (FFPE) tissue specimens for CMS3 and the distinct epigenomic profile in CMS3 [1], we hypothesized that DNA methylation may provide stable and useful markers to discriminate between CMS2 and CMS3. CMS3 tumors exhibit low somatic copy‐number alterations (SCNAs), are hypermutated in 30% of the samples, and have a low number of CpG island methylator phenotype (CIMP) cases with intermediate levels of gene hypermethylation.

Epigenetic gene silencing is one causative factor of CRC development, with DNA methylation as major driving force. Aberrant methylation in cancer is generally characterized by a diffuse DNA hypomethylation and focal hypermethylation in CpG‐rich regions known as CpG islands and their surrounding shores and shelves [9, 10]. CIMP is regarded as a distinct CRC subgroup, which largely overlaps with MSI [11]. Studies suggested that the presence of CIMP plays a role in treatment effect of chemotherapy in patients with stage II/III colon cancer [12, 13]. Furthermore, several DNA methylation biomarkers exhibit high sensitivity and specificity both in detection and in prognosis of CRC [14, 15, 16, 17]. DNA methylation markers are attractive for daily practice due to their stability, and the feasibility to detect these markers in minimally invasive bodily fluids, stool, and FFPE tissue. The aim of this study was to complement currently available CMS classification tools by the identification of a panel of DNA methylation markers to distinguish CMS2 from CMS3 in patients with colorectal cancer.

## 2 Methods

### 2.1 Cohort description

In the MATCH study, a multicentered observational cohort study, fresh‐frozen tumor tissue was collected from stage I‐III colon cancer patients who underwent surgery between 2007 and 2014 in seven hospitals in the Rotterdam region, the Netherlands. Inclusion criteria and additional clinical characteristics of the MATCH study have been described previously [18]. For 239 patients of these patients, matched RNA expression profiles and DNA methylation profiles were generated as described below. The experiments were undertaken with the understanding and written consent of each subject. The study was approved by the Erasmus MC IRB (MEC‐2007‐088), and methodologies conformed to the standards set by the Declaration of Helsinki.

### 2.2 RNA expression profiling and CMS classification

RNA sequencing, data processing, annotation, and normalization of these samples have been described previously [18, 19]. CMS classification was performed on the resulting RNAseq data using the single‐sample prediction parameter from the ‘CMSclassifier’ package (https://github.com/Sage‐Bionetworks/CMSclassifier). Data are available from the European Genome Phenome Archive under accession number EGAS00001002197.

### 2.3 DNA methylation profiling

Genomic DNA was isolated from 30‐µm frozen tissue sections using the NucleoSpin Tissue Kit (Bioké, Leiden, The Netherlands) according to the manufacturer’s instructions. The aforementioned RNA sequencing was performed on the same tissue section. Methylation profiles were generated from 750 ng DNA using the Infinium HumanMethylation450 BeadChip (Illumina, San Diego, CA, USA) according to the manufacturer’s instructions. This platform interrogates over 450 000 methylation sites, covering 99% of all RefSeq genes. Probes have been annotated by Illumina with respect to their position relative to gene regions (within 1500 base pairs (bp) from transcription start site (TSS) (TSS1500), within 200 bp from TSS (TSS200), 5’‐untranslated region (5’UTR), 1st exon, gene body, 3’UTR, or intergenic region, as well as relative to CpG islands (northern shelf (N‐shelf), northern shore (N‐shore), CpG island, southern shore (S‐shore), southern shelf (S‐shelf), or open sea)). Data are available from GSE164811.

### 2.4 Infinium HumanMethylation450 data preprocessing

Raw data were processed and normalized using the Chip Analysis Methylation Pipeline for Illumina HumanMethylation450 and EPIC (ChAMP) package in r [20, 21]. This package contains functions for filtering low‐quality probes, adjustment for Infinium I and Infinium II probe design, batch effect correction, and data normalization. In short, bad‐quality probes (detection *P*‐value > 0.01), probes containing SNPs, probes mapping to multiple locations, and probes mapping to chromosomes X and Y were removed, resulting in 429 705 probes for further analysis. Data were normalized using beta‐mixture quantile (BMIQ) normalization to correct for bias between type I and type II probe chemistry, and potential batch effects were removed using Combat. The returned beta values per probe represent the percentage of methylation for that particular CpG dinucleotide.

### 2.5 Validation data set from TCGA

To validate the analysis results in the MATCH cohort, we used data from The Cancer Genome Atlas (TCGA). Matched RNAseq and Illumina HumanMethylation450 methylation data were available for 274 colorectal carcinomas. For CMS classification of these samples, we again employed the single‐sample prediction parameter from the ‘CMSclassifier’ package (https://github.com/Sage‐Bionetworks/CMSclassifier) to make calls between both cohorts comparable. Resulting single‐sample calls were also compared with the Markov cluster model‐based calls originally reported in the paper by Guinney *et al*. [1] to investigate the effect of using different CMS calling methods.

### 2.6 Data analysis

From the MATCH methylation dataset, we first selected highly variable probes by filtering for probes with a standard deviation of at least 0.15 (beta values) over all samples, which resulted in 52 988 probes (12.3% of all probes in dataset). These probes were matched with TCGA dataset, which contained data for 45 721 of these 52 988 highly variable probes. All subsequent analyses were performed with these 45 721 probes.

#### 2.6.1 Methylation‐level comparisons

To compare overall methylation levels in CMS2 and CMS3 samples, we calculated the median beta value per sample over all 45 721 probes and separately for probes located in (a) CpG islands (19 873 probes), (b) shores (11 111 probes: containing both north and south shores), (c) shelves (2167 probes: containing both north and south shelves), and (d) open sea (12 570 probes). The obtained median methylation values were compared between CMS2 and CMS3 samples using the Wilcoxon rank‐sum test in the MATCH and TCGA dataset separately.

#### 2.6.2 Group‐regularized ridge regression analysis (grridge)

We performed adaptive group‐regularized logistic ridge regression and post hoc group‐weighted elastic net feature selection as described before [22, 23]. Two types of auxiliary data were separately provided to the model for group regularization of the included probes: (a) CpG codata—probe location relative to CpG island (i.e., within a CpG island (CGI), shore (northern and southern combined), shelf (northern and southern combined), or open sea); and (b) CMSori codata—whether the CpG detected by the respective probe was associated with a gene included in the original single‐sample CMS classifier (true for 1637 probes). A regression model was built with the MATCH cohort data using both types of auxiliary data, and 15, 10, and 5 markers were selected by post hoc group‐weighted elastic net feature selection [23]. Performance of the model was first evaluated by 10‐fold cross‐validation in the MATCH cohort. Predicted probabilities for the sample being CMS3 were calculated using the different models. Then, performance of the models was visualized by receiver operating characteristic (ROC) curve and quantified by AUC. Youden’s index was calculated to determine the optimal probability cut off for the 15‐, 10‐, and 5‐marker panels based on the CpG codata and, separately, also for the 15‐, 10‐, and 5‐marker panels based on the CMSori codata. Subsequently, the fixed models were applied to TCGA cohort to validate their performance in an independent dataset. Youden’s index as determined in the MATCH dataset was used as cutoff to determine the sensitivity and specificity of the fixed models in TCGA dataset.

#### 2.6.3 Correlation analysis between DNA methylation and RNA expression

Out of the 45 721 methylation probes used for predictive modeling, 24 904 were located close to a gene’s transcription site (TSS; up to 1500 base pairs (bp) upstream) or within a gene (either in the 5’‐untranslated region (UTR), the gene body, or the 3’UTR). For these probes, we evaluated whether the methylation level we observed in CMS2 and CMS3 samples of the MATCH cohort was associated with RNA expression of the respective gene in the same samples. Spearman’s correlations were calculated for every probe that was matched to a gene, and a false discovery rate (FDR) correction was applied to account for multiple testing.

#### 2.6.4 Multiclass classification

Samples (CMS1‐4) from the MATCH and TCGA cohorts were combined, and a single split was done to obtain a training (*n* = 283) and test (*n* = 141) set. Training and test sets were balanced with respect to CMS class distribution and original cohort. We performed multiclass classification by the sparse group lasso for multinomial response, using r package ‘msgl’ [24], and validated the obtained model from the training set in the test set. To obtain a more balanced representation of the four classes, we double‐weighted the CMS4 samples.

## 3 Results

### 3.1 Cohort description

**Fig. 1:**
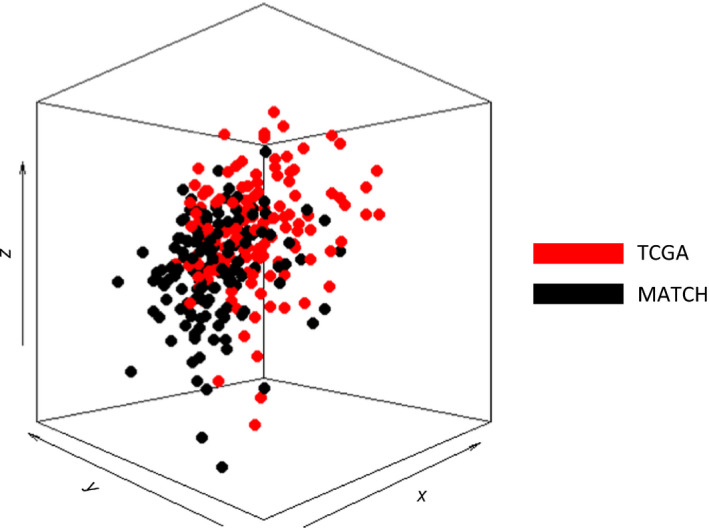
Principal component analysis (PCA) of DNA methylation profiles from all CMS2 and CMS3 samples present in the MATCH and TCGA cohorts. Principal components were calculated for DNA methylation profiles of 286 colorectal cancer tissues (146 from MATCH cohort (black) and 140 from TCGA cohort (red)). PC1, PC2, and PC3 are shown on the *x*‐, *y*, and *z*‐axis, respectively, where each dot represents 1 sample. Samples are colored based on their cohort of origin (MATCH in black and TCGA in red).

Matched RNAseq and Infinium 450K methylation profiles were available for 239 colon cancer patients in the MATCH cohort and 274 colorectal cancer patients in TCGA cohort. Clinical characteristics of both cohorts are shown in Table 1. Differences in pT stage (*P* < 0.001), pN stage (*P* < 0.001), tumor stage (*P* < 0001), tumor location (*P* = 0.023), and CMS classification (*P* = 0.001) were seen between the two cohorts. CMS class was determined on the RNAseq data using the single‐sample predictor, which is independent from other samples. For TCGA cohort, obtained CMS calls with the single‐sample predictor were compared with the original calls from the Markov cluster algorithm [1]. We observed a significant moderate agreement in the CMS calls obtained by the two methods (Cohen’s kappa of 0.51, *P* = 6.92E‐63). However, as shown in Table 2, samples particularly shifted from CMS3 and CMS4 in the Markov cluster algorithm to NA in the single‐sample predictor and from NA in the Markov cluster algorithm to CMS2 in the single‐sample predictor. To ensure that CMS calling was comparable between the MATCH and TCGA cohort, we therefore used the single‐sample predictor calls for both cohorts. Then, CMS2 and CMS3 were selected from the MATCH cohort (124 CMS2 and 22 CMS3) and TCGA cohort (118 CMS2 and 22 CMS3). Within the MATCH cohort, tumor differentiation grade was significantly different between CMS2 and CMS3, and in TCGA cohort, tumor location was significantly different between the two classes (Table S1). Principal component analysis was performed and did not show a strong separation between MATCH and TCGA samples, indicating no obvious bias was introduced by the use of the 2 different cohorts (Fig. 1 and Fig. S1).

**Table 1:** Clinical and histopathological characteristics of all patients.

	MATCH		TCGA		*P*‐value
	*N* = 239	%	*N* = 274	%	
Gender					
Male	126	52.7	146	53.3	0.147
Female	113	47.3	126	46	
Missing			2	0.7	
Age (median, IQR)	68 (61–74)		66 (55–76)		0.674
BMI (median, IQR)	26 (23.5–28.7)				
Tumor stage					
I	62	25.9	44	16.1	< 0.001
II	108	45.2	105	38.3	
III	69	28.9	77	28.1	
IV	0	0	36	13.1	
Missing			12	4.4	
pT stage					
Tis	0	0	1	0.4	< 0.001
1	0	0	7	2.6	
2	70	29.3	41	15	
3	164	68.6	186	67.9	
4	5	2.1	37	13.4	
Missing			2	0.7	
pN stage					
0	171	71.6	160	58.4	< 0.001
1	44	18.4	67	24.5	
2	24	10	45	16.4	
Missing			2	0.7	
Tumor differentiation					
Good	22	9.2			
Moderate	192	80.3			
Poor	19	8			
Unknown/other	6	2.5			
Tumor location					
Right	126	52.7	162	59.1	0.029
Left	113	47.3	95	34.7	
Missing			17	6.2	
Rectum/colon					
Colon	239	100	271	98.9	
Rectum	0	0	1	0.4	
Missing			2	0.7	
Adjuvant therapy					
No	172	72			
Yes	67	28			
CMS					
1	50	20.9	45	16.4	0.001
2	124	51.9	118	43	
3	22	9.2	22	8	
4	8	3.3	35	13.9	
NA	35	14.6	54	19.7	
Microsatellite status					
MSS	180	75.3			
MSI	53	22.2			
Missing	6	2.5			

**Table 2:** CMS calls Markov CLUSTER ALGORITHM vs single‐sample predictor in TCGA dataset.

		CMS single‐sample predictor					Total
		CMS 1	CMS 2	CMS 3	CMS 4	NA	
Markov cluster algorithm	CMS1	31	0	0	0	3	34
	CMS2	0	68	0	0	1	69
	CMS3	1	4	14	0	13	32
	CMS4	1	8	0	34	14	57
	NA	12	38	8	1	23	82
	Total	45	118	22	35	54	274

### 3.2 Comparing CMS2 and CMS3 DNA methylation profiles

**Fig. 2:**
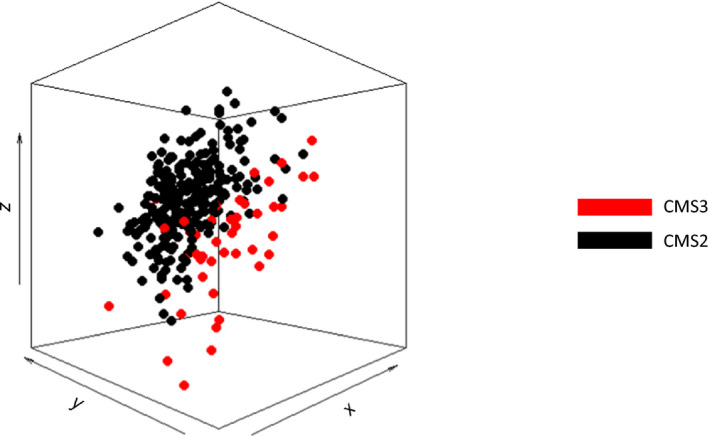
Principal component analysis (PCA) of DNA methylation profiles from all CMS2 and CMS3 samples present in the MATCH and TCGA cohorts. Principal components were calculated for DNA methylation profiles of 286 colorectal cancer tissues (242 CMS2 samples (black) and 44 CMS3 samples). PC1, PC2, and PC3 are shown on the *x*‐, *y*‐, and *z*‐axis, respectively, where each dot represents 1 sample. Samples are colored based on CMS classification (CMS2 in black and CMS3).

**Fig. 3:**
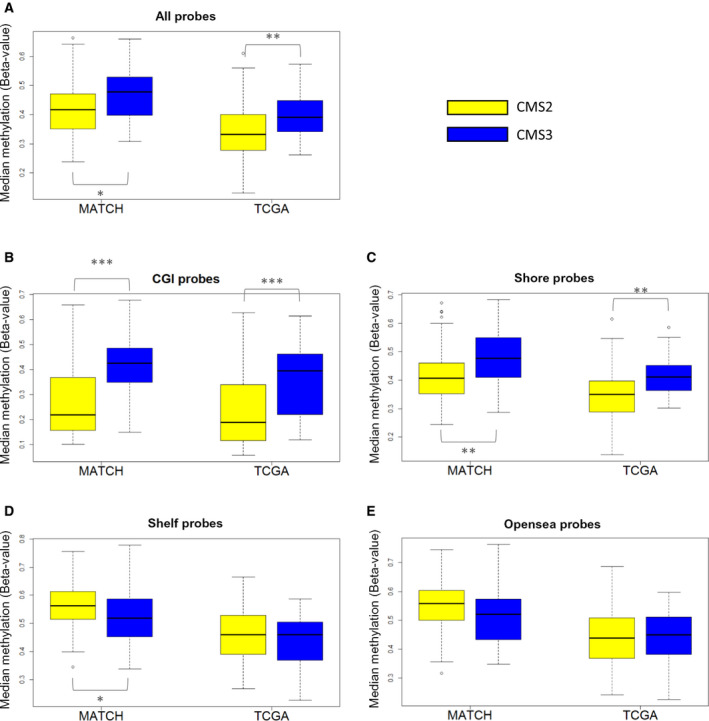
Box plots showing the median methylation levels observed in CMS2 and CMS3 samples where probes are grouped based on their location relative to a CpG island (CGI). These are box‐and‐whisker plots, showing the distribution of the data following the standard conventions; the median as horizontal bar within the box, which depicts the middle 50% of observations. The whiskers extend to 1.5 IQR (interquartile range) below Q1 and above Q3 (lower and upper quartile, respectively). Median methylation levels are shown in CMS2 (white) and CMS3 (gray) samples from the MATCH (left) and TCGA (right) cohorts in A. for all probes included (*n* = 45 721), in B. for probes located in CpG islands (CGI; *n* = 19 837), in C. for probes located in CGI shores (*n* = 11 111), in D. for probes located in CGI shelves (*n* = 2167), and in E. for probes located in the open sea (*n* = 12570). *P < 0.05; **P < 0.01; and ***P < 0.001 (Mann–Whitney *U*‐test).

Principal component analysis of both datasets combined showed that CMS2 and CMS3 samples are partly separated based on overall methylation profiles (Fig. 2 and Fig. S2). Overall, we observed a significantly higher median methylation level for our 45 721 most variable probes in CMS3 compared with CMS2 (Fig. 3A; Mann–Whitney *U*‐test, *P* = 0.012 and 0.004 for MATCH and TCGA, respectively), which is in line with the observations by Guinney *et al*. Interestingly, when we divided probes based on their position relative to CpG islands, a difference between CMS2 and CMS3 was found for those probes located within CpG islands (Fig. 3B; Mann–Whitney *U*‐test, *P* = 2.057E‐5 and 1.750E‐4 for MATCH and TCGA, respectively) or their shores (Fig. 3C; Mann–Whitney U‐test, *P* = 0.005 and 0.002 for MATCH and TCGA, respectively), but not for probes located in shelves (Fig. 3D; Mann–Whitney *U*‐test, *P* = 0.031 and *P* = 0.523 for MATCH and TCGA, respectively) or open sea (Fig. 3E; Mann–Whitney *U*‐test, *P* = 0.104 and 0.964 for MATCH and TCGA, respectively).

### 3.3 Building and validating a prediction model for CMS2 and CMS3 classification

**Fig. 4:**
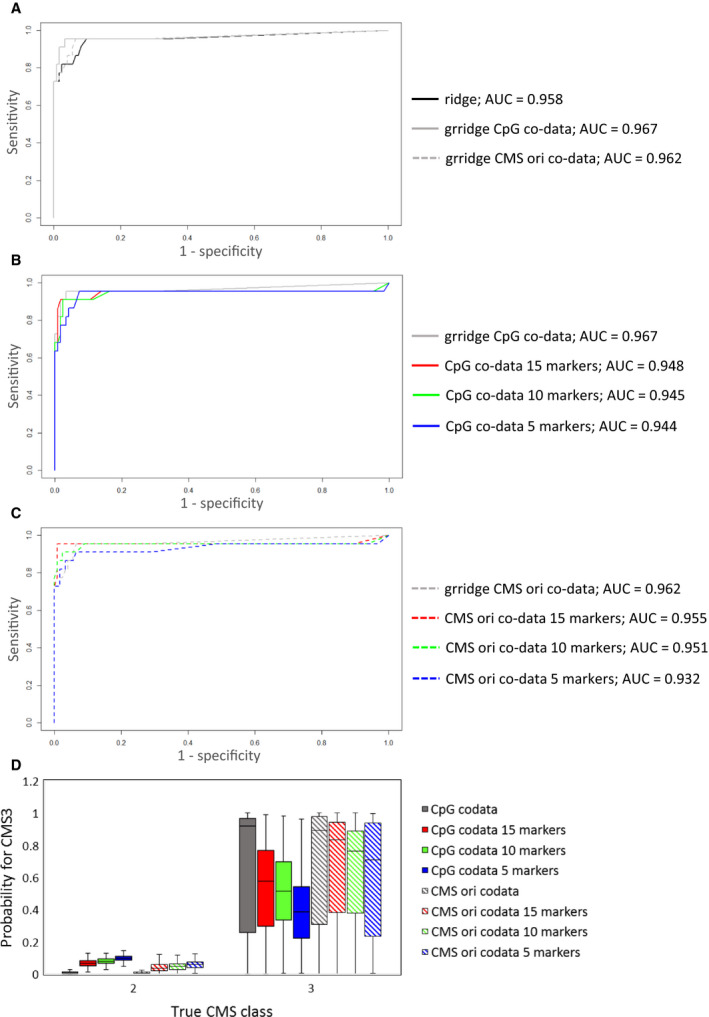
Evaluation of the (gr)ridge prediction models in the training dataset (MATCH). Receiver –operating characteristic (ROC) curves are shown for (A) ordinary ridge (black) and group‐regularized ridge (grridge) models with CpG codata (gray) and CMSori codata (gray dashed line), (B) grridge models based on CpG codata with post hoc group‐weighted elastic net feature selection of 15 (red), 10 (green), and 5 (blue) markers, and (C) grridge models based on CMSori codata with post hoc group‐weighted elastic net feature selection of 15 (red), 10 (green), and 5 (blue) markers. In (D) the obtained probabilities for CMS3 are plotted for CpG codata (solid fill) and CMSori codata (striped fill) models with all features (gray), 15 markers (red), 10 markers (green), and 5 markers (blue).

Next, we used the MATCH dataset to build a prediction model for the classification of CMS2 and CMS3, using group‐regularized logistic ridge regression (grridge) and group‐weighted post hoc feature selection [22, 23]. As shown in Fig. 4A, the ordinary ridge algorithm already performed well in the classification of CMS2 versus CMS3. Group regularization of the probes, based on either their relative location to a CpG island (CpG codata panel) or their location relative to genes in the original CMS classifier (CMSori codata panel), improved the AUC by only 1% or 0.5%, respectively. Group‐weighted feature selection down to 15, 10, and 5 markers yielded largely different marker panels for both types of codata used (4 overlapping markers; Table 3) that still performed well in the classification (Fig. 4B,C). However, the obtained probabilities for CMS3 increased in the true CMS2 samples, whereas they decreased in the true CMS3 samples when the number of markers was reduced (Fig. 4D). The methylation levels of all selected probes for classification between CMS2 and CMS3 are depicted in Fig. S3A (MATCH cohort) and Fig. S3B (TCGA cohort).

**Table 3:** Selected probes.

probe_ID	Gene	chr	Position (bp)	Gene‐CpG	CpG codata			CMSori codata		
					15m	10m	5m	15m	10m	5m
cg19335412	ACTA2	10	90694875	3'UTR‐open sea				+		
cg23928468	SLC5A6	2	27433191	5'UTR‐shore				+		
cg05951860	CTTNBP2	7	117513101	Body‐island				+		
cg20698769	CTTNBP2	7	117513002	Body‐island				+	+	+
cg17477990	PDE4DIP	1	144937317	Body‐open sea	+	+	+			
cg11125249	GYG1	3	148737622	Body‐open sea	+					
cg02827572	C6orf106	6	34566245	Body‐open sea	+					
cg04739880	ANKS1A	6	35017865	Body‐open sea	+	+				
cg00512872	CYTH3	7	6268584	Body‐open sea	+	+	+			
cg14754494	DDC	7	50560743	Body‐open sea	+	+		+	+	+
cg19107055	DDC	7	50560686	Body‐open sea	+	+	+	+	+	+
cg05357660	PREP	6	105750581	Body‐open sea				+	+	
cg23045908	PDE4B	1	66799419	Body‐open sea				+		
cg16708174	RARRES1	3	158430962	Body‐open sea				+	+	
cg00901138	CHN2	7	29329370	Body‐open sea				+	+	
cg00901574	POFUT1	20	30804997	Body‐open sea				+		
cg16477879	ASB1	2	239348171	Body‐shelf	+	+	+			
cg23219253	ASAP2	2	9518751	Body‐shelf	+	+	+			
cg05211192	MAD1L1	7	2119076	Body‐shelf	+					
cg12492273	MAD1L1	7	2119499	Body‐shelf	+					
cg16772998	MAD1L1	7	2119116	Body‐shelf	+	+				
cg00145955	QPRT	16	29703480	Body‐shelf	+	+		+	+	+
cg00097384	QPRT	16	29703459	Body‐shelf				+	+	
cg27603796	CTTNBP2	7	117512803	Body‐shore				+	+	
cg23418465		3	126239121	IGR‐shelf	+					
cg17842966	FCGBP	19	40441469	TSS1500‐open sea	+	+		+	+	+

Youden’s index was calculated to determine the optimal cutoff for the marker panels based on either the CpG codata and or the CMSori codata separately. Even when only 5 markers are used, sensitivities, specificities and accuracies > 90% are observed for both codata marker panels in the MATCH dataset using Youden’s index as a cutoff (Table 4).

**Table 4:** Performance of both marker panels in MATCH cohort and TCGA cohort.

No of probes	Model used	CpG		CMSori	
	Dataset	MATCH	TCGA	MATCH	TCGA
15	Cut off (Youden's index in MATCH data)	0.24/0.25	0.16		
	TNR (spec)	0.98	0.99	0.99	0.98
	TPR (sens)	0.91	0.82	0.95	0.95
	Accuracy	0.97	0.96	0.99	0.98
10	Cut off (Youden's index in MATCH data)	0.21/0.22	0.13		
	TNR (spec)	0.98	0.98	0.98	0.94
	TPR (sens)	0.91	0.82	0.91	0.95
	Accuracy	0.97	0.96	0.97	0.94
5	Cut off (Youden's index in MATCH data)	0.14	0.11		
	TNR (spec)	0.93	0.92	0.94	0.92
	TPR (sens)	0.95	0.91	0.91	0.91
	Accuracy	0.93	0.91	0.93	0.92

The obtained models were subsequently fixed and applied to the CMS2 and CMS3 samples from TCGA dataset, to verify the models’ efficacy to predict CMS3 status in independent samples. Using the optimal cutoffs selected in the MATCH dataset, the highest performance was established with the 15 marker panels. Again, even the 5‐marker panels yielded sensitivities, specificities and accuracies > 90% in TCGA dataset as well (Table 4).

### 3.4 Correlation between DNA methylation levels and RNA expression

To determine the potential impact of the observed methylation patterns on gene expression, we calculated the Spearman correlation between DNA methylation levels and RNA expression for all gene‐associated methylation probes in the MATCH cohort. In total, Spearman’s correlations were determined for 24 904 probes. Of these probes, 10.9% and 25.6% were significantly positively and negatively correlated with the expression of their associated gene, respectively. Together, our marker panels included 26 probes of which 25 were associated with a gene. We observed that 28% and 36% were significantly positively and negatively correlated to the expression of their associated gene, respectively (Table 5). For the CMSori codata marker panel, selection of gene‐associated probes was favored by the codata itself (probes associated with genes included in the CMS SSP classifier), which resulted in 26.7% of the markers showing positive correlation and 53.3% showing negative correlation. In contrast, for the CpG codata marker panel we observed that 35.7% of gene‐associated markers were positively correlated with expression, whereas only 14.3% were negatively correlated. Compared with all probes (*n* = 24879) not included in our marker panels, we found that the CpG codata marker panel was significantly enriched for positively correlated probes (chi‐square test, *P* = 0.003), whereas the CMSori codata marker panel was significantly enriched for positively and negatively correlated probes (chi‐square test, *P* = 0.049 and *P* = 0.014, respectively).

**Table 5:** Correlation between methylation levels and expression levels.

Probe	Gene‐CpG	ENSG	Gene symbol	CpG codata	CMSori codata	Spearman's Rho	FDR
cg23928468	5'UTR‐shore	ENSG00000138074	SLC5A6	No	Yes	−0.56	2.83E‐12
cg00901574	Body‐open sea	ENSG00000101346	POFUT1	No	Yes	−0.55	1.34E‐11
cg00512872	Body‐open sea	ENSG00000008256	CYTH3	Yes	No	0.47	3.64E‐08
cg17842966	TSS1500‐open sea	ENSG00000275395	FCGBP	Yes	Yes	−0.44	2.16E‐07
cg00097384	Body‐shelf	ENSG00000103485	QPRT	No	Yes	0.42	1.67E‐06
cg00145955	Body‐shelf	ENSG00000103485	QPRT	Yes	Yes	0.40	4.44E‐06
cg27603796	Body‐shore	ENSG00000077063	CTTNBP2	No	Yes	−0.34	1.23E‐04
cg16708174	Body‐open sea	ENSG00000118849	RARRES1	No	Yes	−0.34	1.43E‐04
cg20698769	Body‐island	ENSG00000077063	CTTNBP2	No	Yes	−0.34	1.91E‐04
cg23045908	Body‐open sea	ENSG00000184588	PDE4B	No	Yes	−0.30	1.15E‐03
cg04739880	Body‐open sea	ENSG00000064999	ANKS1A	Yes	No	0.27	3.76E‐03
cg19107055	Body‐open sea	ENSG00000132437	DDC	Yes	Yes	0.25	9.67E‐03
cg17477990	Body‐open sea	ENSG00000178104	PDE4DIP	Yes	No	−0.24	1.42E‐02
cg00901138	Body‐open sea	ENSG00000106069	CHN2	No	Yes	0.24	1.46E‐02
cg05951860	Body‐island	ENSG00000077063	CTTNBP2	No	Yes	−0.21	3.06E‐02
cg16477879	Body‐shelf	ENSG00000065802	ASB1	Yes	No	0.21	3.42E‐02
cg19335412	3'UTR‐open sea	ENSG00000107796	ACTA2	No	Yes	0.14	1.70E‐01
cg12492273	Body‐shelf	ENSG00000002822	MAD1L1	Yes	No	0.14	1.96E‐01
cg23219253	Body‐shelf	ENSG00000151693	ASAP2	Yes	No	−0.13	2.30E‐01
cg14754494	Body‐open sea	ENSG00000132437	DDC	Yes	Yes	0.11	3.10E‐01
cg11125249	Body‐open sea	ENSG00000163754	GYG1	Yes	No	−0.05	7.09E‐01
cg16772998	Body‐shelf	ENSG00000002822	MAD1L1	Yes	No	−0.05	7.12E‐01
cg02827572	Body‐open sea	ENSG00000196821	C6orf106	Yes	No	−0.04	7.40E‐01
cg05211192	Body‐shelf	ENSG00000002822	MAD1L1	Yes	No	0.04	7.41E‐01
cg05357660	Body‐open sea	ENSG00000085377	PREP	No	Yes	−0.01	9.32E‐01

### 3.5 A DNA methylation‐based multiclass CMS prediction model

Although dedicated assays are already available for CMS1 and CMS4, ideally one would prefer to have one affordable and practical CMS classification assay applicable to FFPE. Therefore, we also evaluated the potential of DNA methylation for multiclass prediction of CMS1‐4. For this purpose, the MATCH and TCGA datasets were combined and split into a training (*n* = 283) and test (*n* = 141) set with balanced CMS class distributions and equal contributions from both cohorts. Results obtained applying the model from the training set to the test set indicate that CMS1, CMS2, and CMS3 can be reliably distinguished based on their DNA methylation profiles (Table 6). CMS4, however, is frequently misclassified as CMS2. Using TCGAbiolinks [25], we found that in TCGA dataset the estimated tumor purity was significantly lower in CMS4 cases, suggesting a larger stromal contribution in these samples (Kruskal–Wallis test, *P* = 1.30E‐31) [26], which may partly explain the classification difficulties. Together, these results indicate that DNA methylation markers are not able to reliably classify colon cancers as CMS4 and that the already described dedicated IHC and qRT‐PCR assays appear better suited for this purpose [7, 8].

**Table 6:** Classification of CMS 1‐4 based on DNA methylation profiles.

		True CMS class				Total
		CMS 1	CMS2	CMS3	CMS4	
Predicted	CMS1	29	0	2	1	32
	CMS2	1	78	2	7	88
	CMS3	1	0	10	0	11
	CMS4	1	2	0	7	10
	Total	32	80	14	15	141
	Correct (%)	90.63	97.50	71.43	46.67	
	False (%)	9.38	2.50	28.57	53.33	

## 4 Discussion

In this study, we aimed to identify DNA methylation markers to distinguish between CMS2 and CMS3 in patients with primary CRC based on a genome‐wide analysis of DNA methylation in fresh‐frozen tumor tissues. We showed that CMS2 and CMS3 samples can be distinguished based on overall methylation profiles using subsequent principal component analysis of two independent datasets, and these datasets combined. Group regularization of the methylation probes was done based on their location either relative to a CpG island or relative to a gene present in the CMS classifier. This resulted in two different prediction models and subsequently different marker panels. For both panels, even when using only 5 markers, the sensitivity, specificity, and accuracy were > 90%. Independent validation of the fixed models in TCGA data showed equal performances. Exploratory multiclass prediction analyses indicate that CMS4 cases are often misclassified as CMS2 based on their DNA methylation profiles.

Thus far, almost all CRC subtyping studies were based on fresh tissue samples, and it remains questionable whether this classification is readily applicable to other types of specimens that are available in the clinic. FFPE‐derived RNA is highly degraded and chemically modified, which can impact its utility as a faithful source for classification. Also for the CMS, previous studies have shown that the CMS classifier developed by Guinney *et al*. had a poor performance in FFPE and on biopsy specimen, especially for CMS3 with a specificity of 0.70 [1, 27]. This high type II error rate in CMS3 suggests either biological or technical differences between FFPE and fresh‐frozen samples and emphasizes the importance of using FFPE samples for training a classifier in this context. Other previous studies have performed DNA methylation analysis of FFPE tissues and provided promising results for the use of FFPE material for DNA methylation profiling [28, 29, 30, 31]. Therefore, in contrast to an RNA‐based classifier, the methylation panel created in this study is likely to work well on FFPE and may thus provide a promising alternative for use in daily clinical practice.

Correlation analysis has been widely used to examine the relationship between methylation and gene expression. Several studies have elucidated hypermethylation of CpG islands at promoter regions, which represses transcription of tumor suppressor genes [32]. However, only one of the probes we identified in both panels was located in the promotor region (within 1500 bp upstream of TSS) of the nearby gene, whereas, except for one intergenic probe, all other probes were located in gene bodies. This is in line with previous research, which showed the impact of DNA methylation at intergenic regions and gene bodies on gene expression [33, 34]. DNA methylation in gene body CpG islands shows an apparent intriguing positive correlation between methylation and gene expression [35, 36]. Yang *et al*. found that from the large amount of methylated probes found in gene body regions, about 20% exhibit a positive correlation between DNA methylation and gene expression. A large proportion of these positively correlated genes were overexpressed in primary colon cancer samples compared with normal colon tissues. Our study shows similar results with 28% of the probes from both marker panels being significantly positively correlated to expression of their associated genes. These findings combined highlight the importance of methylation in gene bodies and warrant further research. Furthermore, our results show that a difference exists between levels of methylation in CMS2 and CMS3 regarding the position of probes relative to CpG islands. This difference was found for methylation of probes within CpG island and shores, but not for probes located in shelves or open sea. This is in line with previous research, which shows that most tissue‐specific DNA methylation and cancer‐specific DNA methylation occur at CpG island shores, especially for colon cancer [37].

Despite the observation that methylation levels in CMS3 were higher in CpG islands and shores compared with CMS2, probes selected by the grridge algorithm as discriminatory panel between CMS2 and CMS3 were actually located in CpG island shelves and in the open sea and mostly showed lower methylation levels in CMS3.

Interestingly, the CMSori codata marker panel was enriched for both positively and negatively correlated probes compared with all probes not selected in the panel. This suggests that DNA methylation is at least partly underlying the expression patterns used for the original CMS2 and CMS3 classification. From the selected probes for which methylation and expression were significantly correlated, *DDC* expression levels were previously described to vary among colorectal cancer tissues and were associated with disease‐free and overall survival [38]. Downregulation of *FCGBP* has been described as a potential target for identification of CRC, and lower expression levels were also associated with poorer survival within CRC patients [39]. *POFUT1* expression was associated with Notch signaling and decreased goblet cell differentiation and was identified as a potential driver of tumor progression in colorectal adenomas [40]. *PDE4B*, which regulates cellular cAMP concentrations, plays a significant role in regulating the malignant phenotype of CRC cells [41]. *RARRES1* is among the most commonly methylated genes and is silenced in multiple cancers. Interestingly, it is also differentially expressed in metabolism‐associated diseases [42], supporting a potential role in CMS3, which is featured as the metabolic subtype.

The CMS classification revealed a relatively large number of CMS2 cases and low number of CMS3 cases in the present series. Taking into account the different sample sizes of this study and the original CMS publication, and given the variation in distributions of CMS classes among the six datasets from which the CMS classification originated [1, 7, 43, 44, 45, 46, 47, 48, 49], it may be that the CMS class distribution varies per dataset. We chose to use the SSP method for classification because it is not sensitive to the composition of the dataset to which it is applied, so the context of a large series of CRCs or batch effect removal is not required.

Previous literature already provides support for the predictive value of CMS [2]. In addition, new prospective clinical studies are being performed to investigate whether CMS classification can indeed be of added value in clinical decision making by analyzing its predictive value for chemotherapy response [50, 51]. In the future, treatments for colon cancer patients will likely be subtype‐specific by targeting characteristically overexpressed molecular targets per consensus subgroup [52]. Therefore, a practical, minimally invasive test to distinguish between the subtypes is needed. Our results show that DNA methylation profiles can be used to discriminate between CMS1, CMS2, and CMS3 cases but do not allow for reliable classification of CMS4. This may due to the relatively large stromal contribution to the CMS4 signature, which is not captured very well in the DNA methylation profile due to the low cell density of stroma. In addition, even though DNA methylation can be used to classify CMS1, we feel that MSI testing, already implemented in routine diagnostics, is more relevant and will capture the vast majority of CMS1 cases [1, 4, 5, 6].

## 5 Conclusion

For future studies and retrospective analyses of archival cohorts, our methylation marker panel should enable the development of a qPCR DNA methylation assay for distinguishing CMS2 from CMS3 in patients with CRC. Such an assay can provide a specific, convenient, and easily implementable tool for use in routine diagnostics. Combined with the already‐developed assays for CMS1 and CMS4, this assay may accelerate the evaluation of the clinical value of CMS classification and will ultimately help physicians in selecting patients for adjuvant treatment based on their CMS classification.

## Funding

This research was funded by the Dutch Cancer Society (KWF), grant number 2013‐6331.

## Conflict of interest

The authors declare no conflict of interest.

## Author contributions

IB conceptualized the study, performed formal analysis and investigation, wrote the original draft, and performed visualization. MS conceptualized the study, performed methodology, validation, formal analysis, and investigation, and wrote, reviewed, and edited the manuscript. RRJC conceptualized the study, provided resources, and wrote, reviewed, and edited the manuscript. MAW contributed to methodology and software, and wrote, reviewed, and edited the manuscript. CHMD contributed to validation and investigation, and wrote, reviewed, and edited the manuscript. VW performed validation and investigation, and wrote, reviewed, and edited the manuscript. JWMM conceptualized the study, provided resources, wrote reviewed, and edited the manuscript, and underwent supervision. JNMI conceptualized the study, provided resources, wrote, reviewed, and edited the manuscript, and underwent supervision. SMW conceptualized the study, contributed to methodology, validation, and investigation, wrote the original draft, and performed visualization and supervision.

### Peer Review

The peer review history for this article is available at https://publons.com/publon/10.1002/1878‐0261.13098.

## Supporting information


**Fig. S1**. Principal Component Analysis (PCA) of DNA methylation profiles from all CMS2 and CMS3 samples present in the MATCH and TCGA cohorts.

**Fig. S2**. Principal Component Analysis (PCA) of DNA methylation profiles from all CMS2 and CMS3 samples present in the MATCH and TCGA cohorts.

**Fig. S3A**. Boxplots of methylation levels for all selected markers in the MATCH cohort.

**Fig. S3B**. Boxplots of methylation levels for all selected markers in the TCGA cohort.

**Table S1**. Clinical and histopathological characteristics of CMS2 and CMS3 patients per cohort.

## Data Availability

The data that support the findings of this study are available from the corresponding author upon reasonable request.
