# Supplementary material for: A panel of DNA methylation markers for the classification of consensus molecular subtypes 2 and 3 in patients with colorectal cancer
Source: Mol Oncol. 2021 Sep 30;15(12):3348–62. doi: 10.1002/1878-0261.13098 (PMC8637568; doi:10.1002/1878-0261.13098)
Supplement: Supplementary file 2 — Fig. S2. Principal Component Analysis (PCA) of DNA methylation profiles from all CMS2 and CMS3 samples present in the MATCH and TCGA cohorts. [file MOL2-15-3348-s005.pptx]

## Slide 1
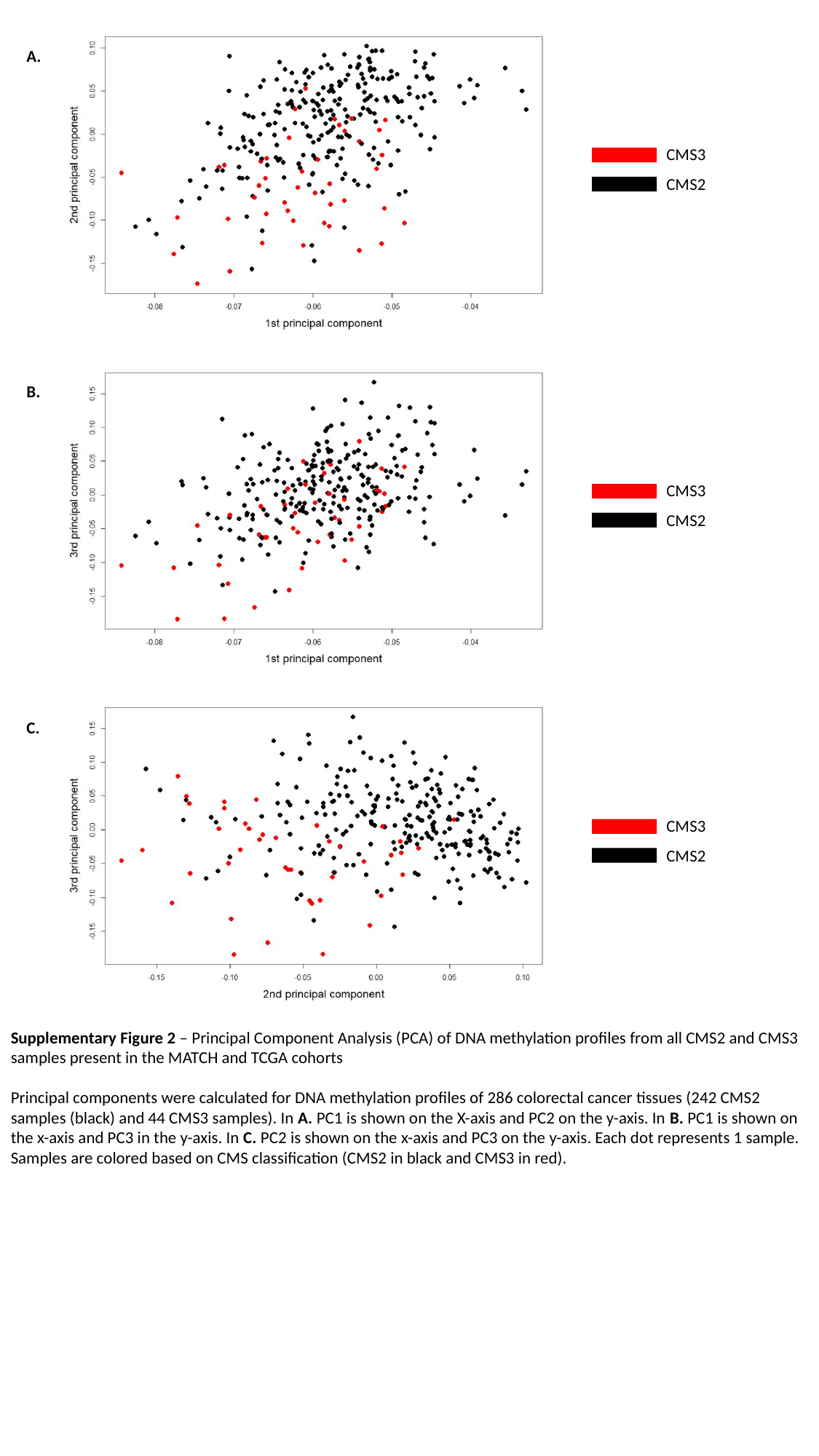

A.
CMS3
CMS2
B.
CMS3
CMS2
C.
CMS3
CMS2
Supplementary Figure 2 – Principal Component Analysis (PCA) of DNA methylation profiles from all CMS2 and CMS3 samples present in the MATCH and TCGA cohorts
Principal components were calculated for DNA methylation profiles of 286 colorectal cancer tissues (242 CMS2 samples (black) and 44 CMS3 samples). In A. PC1 is shown on the X-axis and PC2 on the y-axis. In B. PC1 is shown on the x-axis and PC3 in the y-axis. In C. PC2 is shown on the x-axis and PC3 on the y-axis. Each dot represents 1 sample. Samples are colored based on CMS classification (CMS2 in black and CMS3 in red).
