# Supplementary material for: A panel of DNA methylation markers for the classification of consensus molecular subtypes 2 and 3 in patients with colorectal cancer
Source: Mol Oncol. 2021 Sep 30;15(12):3348–62. doi: 10.1002/1878-0261.13098 (PMC8637568; doi:10.1002/1878-0261.13098)
Supplement: Supplementary file 3 — Fig. S3A. Boxplots of methylation levels for all selected markers in the MATCH cohort. [file MOL2-15-3348-s001.pdf]

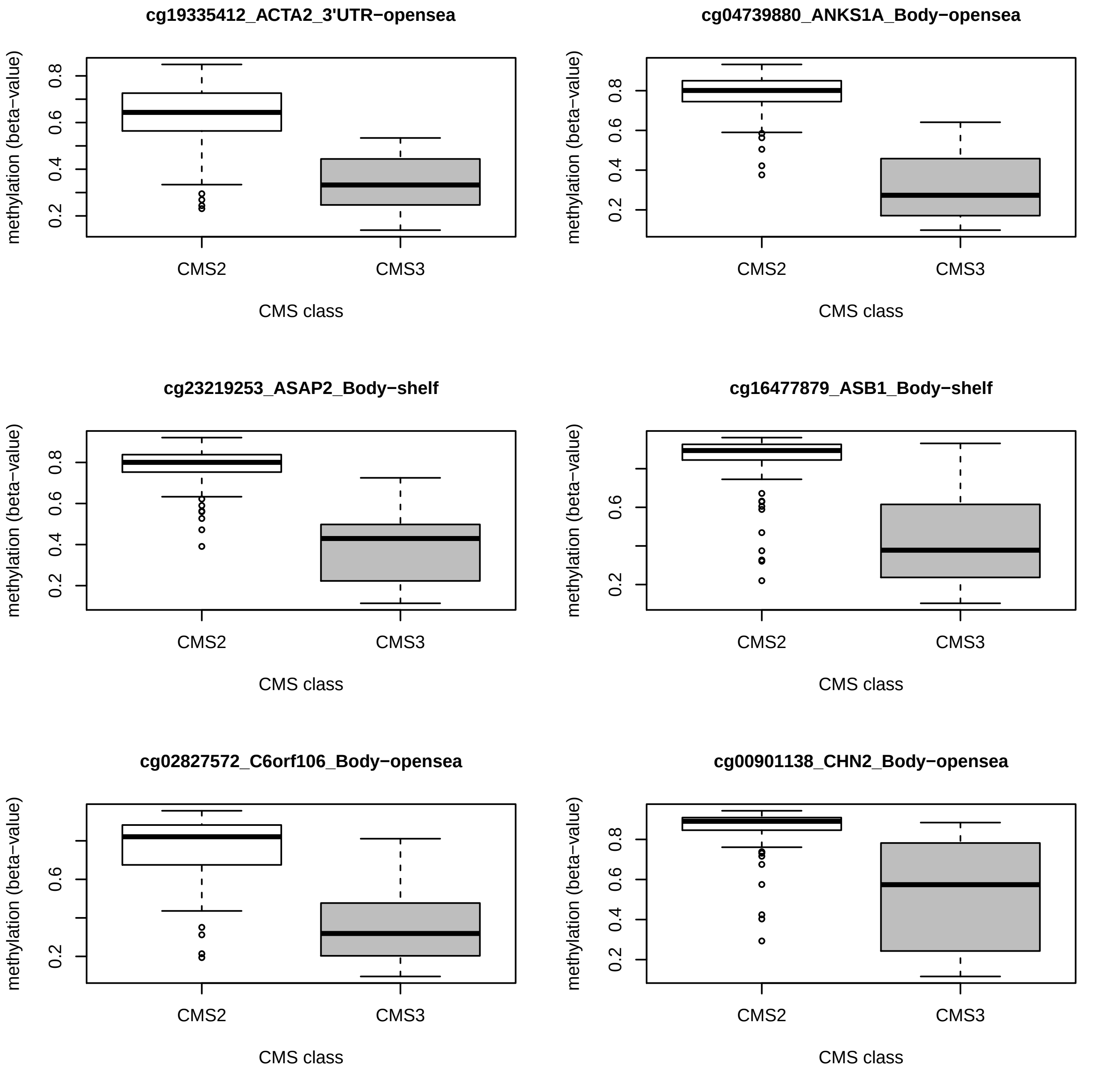
cg19335412_ACTA2_3'UTR−opensea
cg04739880_ANKS1A_Body−opensea
cg23219253_ASAP2_Body−shelf
cg16477879_ASB1_Body−shelf
cg02827572_C6orf106_Body−opensea
cg00901138_CHN2_Body−opensea
methylation (beta−value)
0.2
0.4
0.6
0.8
CMS2
CMS3
CMS class

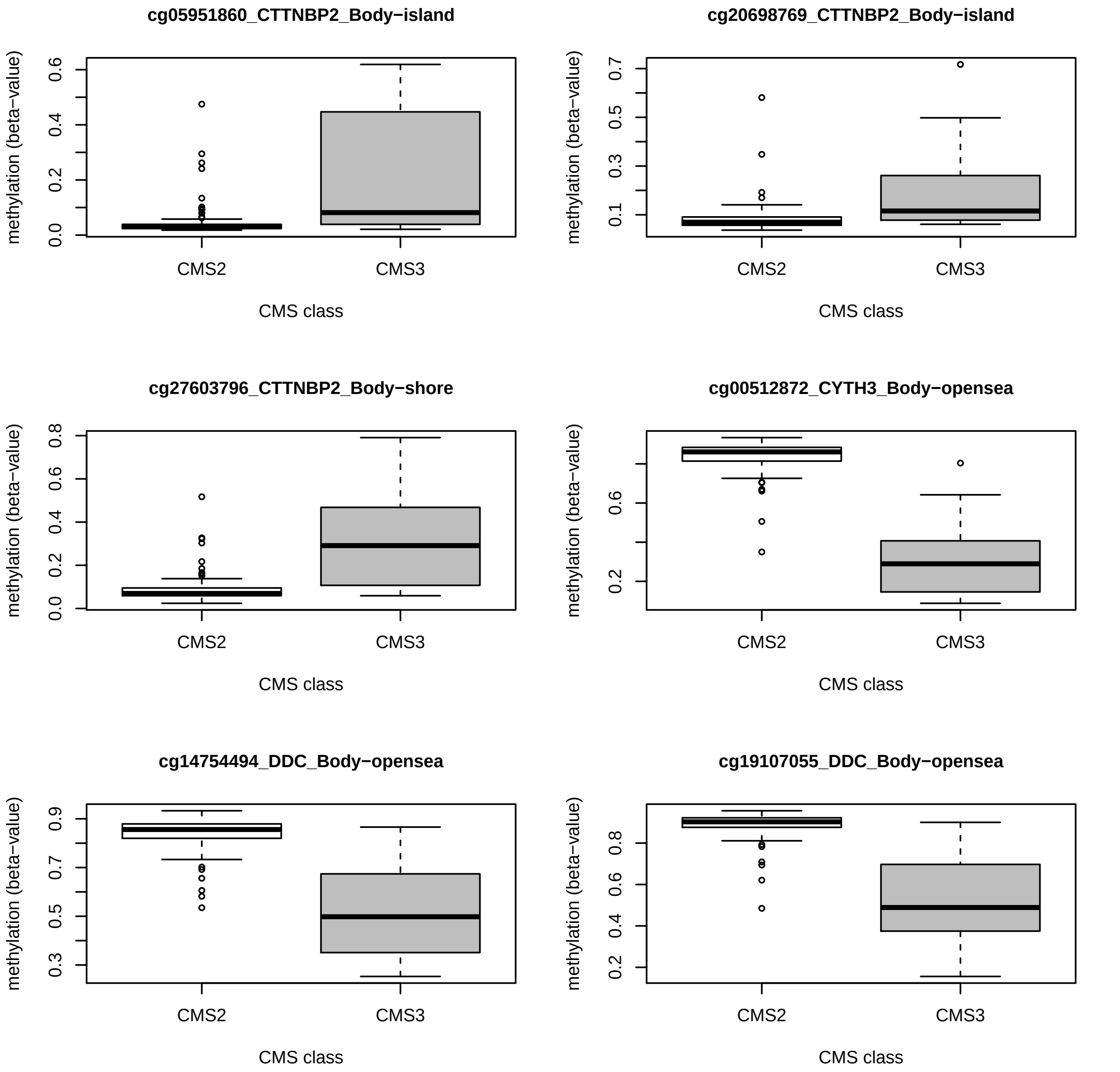
cg05951860_CTTNBP2_Body−island
methylation (beta−value)
0.0
0.2
0.4
0.6
CMS2
CMS3
CMS class
cg20698769_CTTNBP2_Body−island
methylation (beta−value)
0.1
0.3
0.5
0.7
CMS2
CMS3
CMS class
cg27603796_CTTNBP2_Body−shore
methylation (beta−value)
0.0
0.2
0.4
0.6
0.8
CMS2
CMS3
CMS class
cg00512872_CYTH3_Body−opensea
methylation (beta−value)
0.2
0.6
CMS2
CMS3
CMS class
cg14754494_DDC_Body−opensea
methylation (beta−value)
0.3
0.5
0.7
0.9
CMS2
CMS3
CMS class
cg19107055_DDC_Body−opensea
methylation (beta−value)
0.2
0.4
0.6
0.8
CMS2
CMS3
CMS class

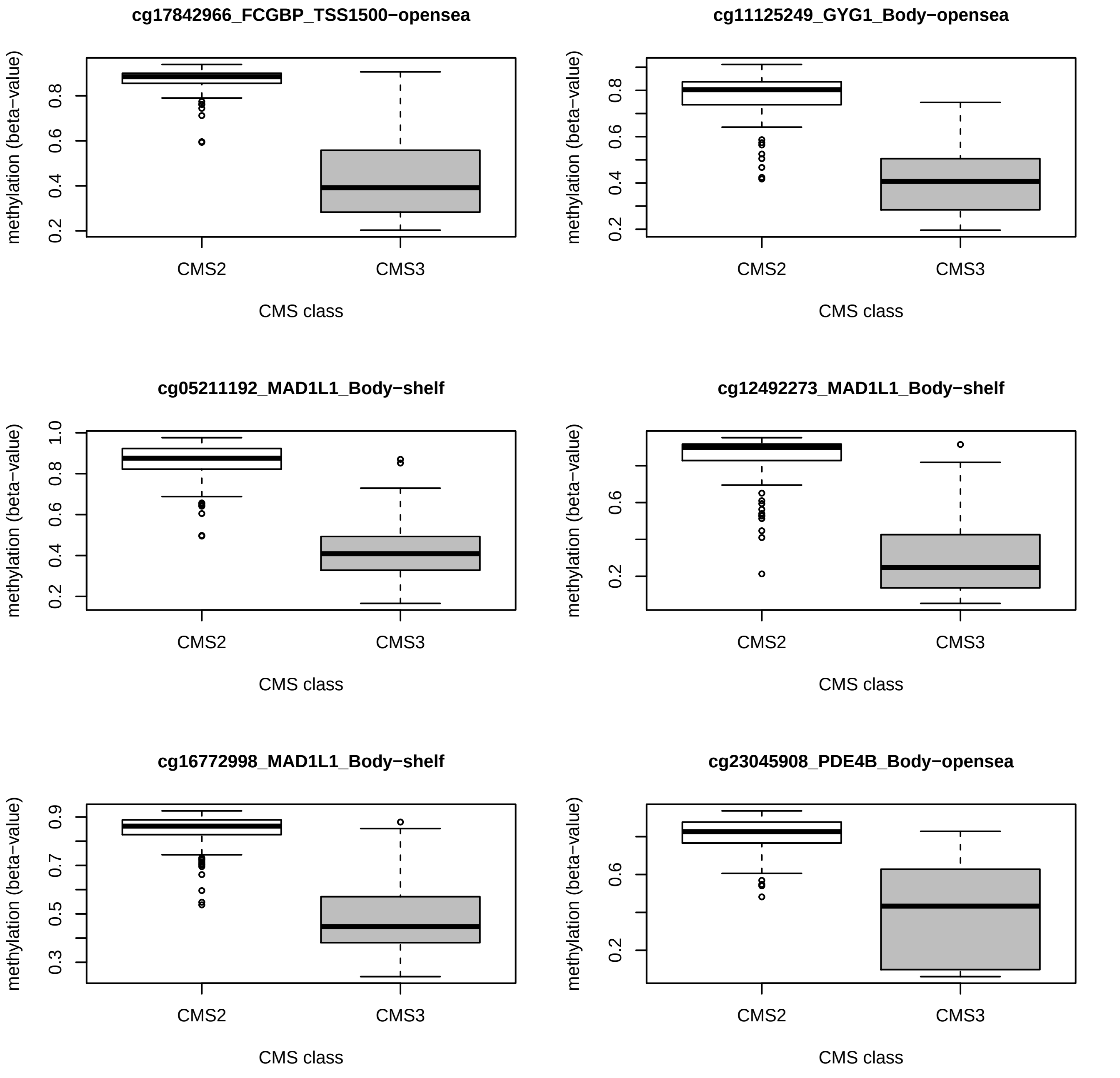
cg17842966_FCGBP_TSS1500−opensea
methylation (beta−value)
0.2
0.4
0.6
0.8
CMS2
CMS3
CMS class
cg11125249_GYG1_Body−opensea
methylation (beta−value)
0.2
0.4
0.6
0.8
CMS2
CMS3
CMS class
cg05211192_MAD1L1_Body−shelf
methylation (beta−value)
0.2
0.4
0.6
0.8
1.0
CMS2
CMS3
CMS class
cg12492273_MAD1L1_Body−shelf
methylation (beta−value)
0.2
0.6
CMS2
CMS3
CMS class
cg16772998_MAD1L1_Body−shelf
methylation (beta−value)
0.3
0.5
0.7
0.9
CMS2
CMS3
CMS class
cg23045908_PDE4B_Body−opensea
methylation (beta−value)
0.2
0.6
CMS2
CMS3
CMS class

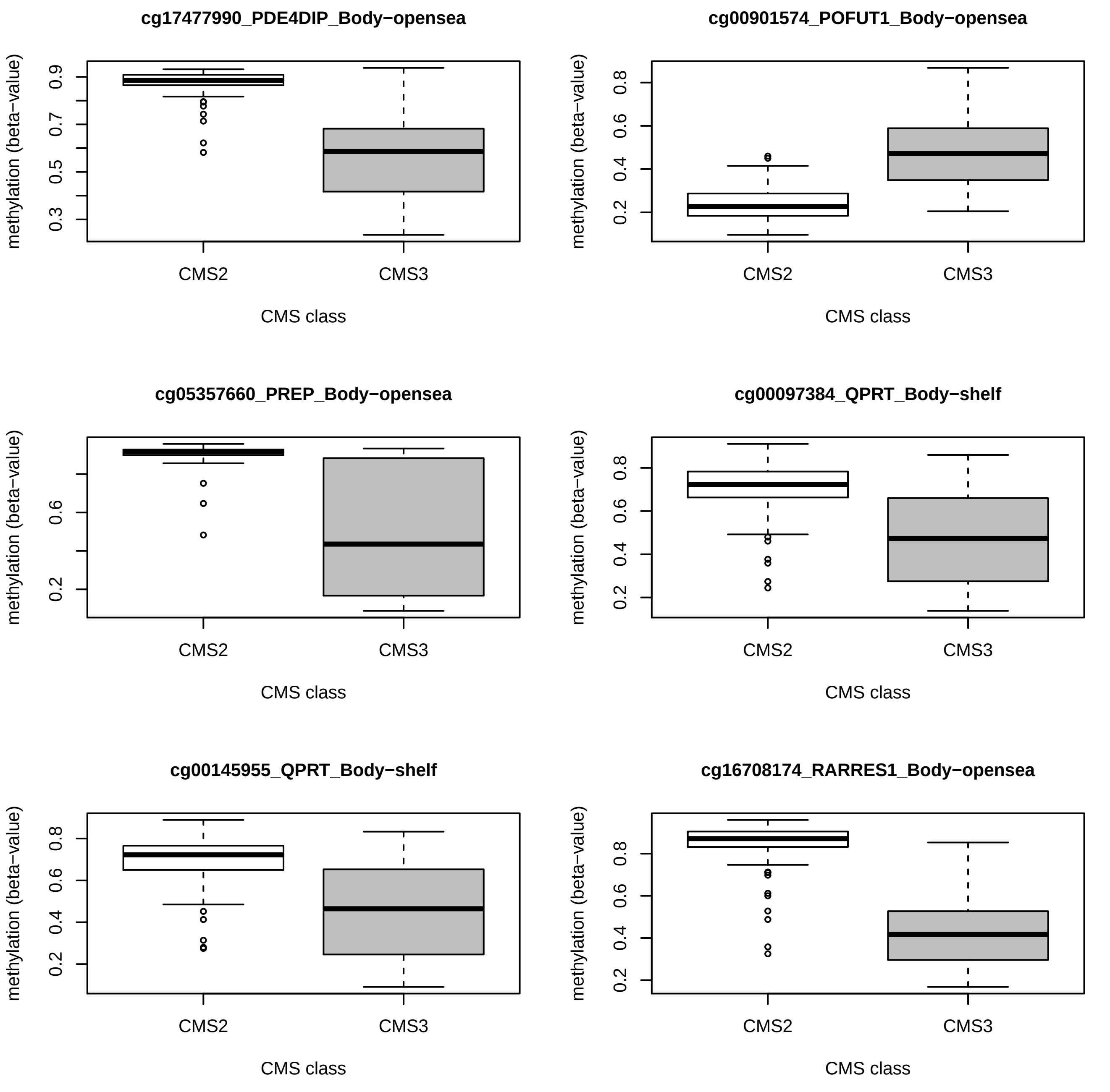
cg17477990_PDE4DIP_Body−opensea
cg00901574_POFUT1_Body−opensea
cg05357660_PREP_Body−opensea
cg00097384_QPRT_Body−shelf
cg00145955_QPRT_Body−shelf
cg16708174_RARRES1_Body−opensea
methylation (beta−value)
CMS2
CMS3
CMS class
0.3
0.5
0.7
0.9
0.2
0.4
0.6
0.8

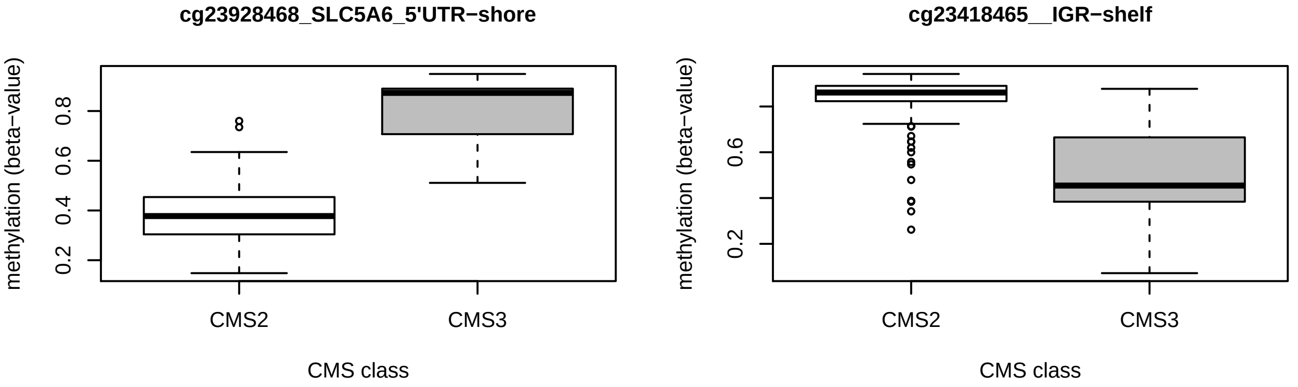

Supplementary Figure 3A - Boxplots of methylation levels for all selected markers in the MATCH cohort.

Methylation levels for all 26 selected markers are shown for CMS2 (white box) and CMS3 (grey box) samples from the MATCH cohort. Boxes represent the interquartile range (IQR; 25th - 75th percentile), whereas the black line indicates the median. Whiskers show the extremes (1.5*IQR above the 75th and 1.5*IQR below the 25th percentile, respectively). Outlier samples are depicted as open circles.
