# Supplementary material for: A panel of DNA methylation markers for the classification of consensus molecular subtypes 2 and 3 in patients with colorectal cancer
Source: Mol Oncol. 2021 Sep 30;15(12):3348–62. doi: 10.1002/1878-0261.13098 (PMC8637568; doi:10.1002/1878-0261.13098)
Supplement: Supplementary file 5 — Table S1. Clinical and histopathological characteristics of CMS2 and CMS3 patients per cohort. [file MOL2-15-3348-s004.docx]

| ***Supplementary Table 1. Clinical and histopathological characteristics of CMS2 and CMS3 patients per cohort*** | | | | | | | | | | | | | | |
| --- | --- | --- | --- | --- | --- | --- | --- | --- | --- | --- | --- | --- | --- | --- |
|  | **Match** | | | | | | | **TCGA** | | | | | | |
|  | **Total** | **(%)** | **CMS 2** | (%) | **CMS 3** | (%) | ***p*-value** | **Total** | **(%)** | **CMS 2** | **(%)** | **CMS 3** | **(%)** | ***p*-value** |
|  | **N = 146** |  | **N = 124** | **84.9** | **N = 22** | 1**5.1** |  | **N = 140** |  | **N = 118** | **(84.3)** | **N = 22** | **15.7** |  |
| **Gender** |  |  |  |  |  |  | 0.642 |  |  |  |  |  |  | 0.810 |
| Male | 87 | (40.4) | 49 | (39.5) | 10 | (45.5) |  | 78 | (55.7) | 66 | (55.9) | 10 | (45.5) |  |
| Female | 59 | (59.6) | 75 | (60.5) | 12 | (54.5) |  | 60 | (42.9) | 50 | (42.4) | 12 | (54.5) |  |
|  |  |  |  |  |  |  |  | 2 | (1.4) | 2 | (1.7) | 0 | (0) |  |
| **Age** (median. IQR) | 67 (60-74) | | 67 (60 – 74) | | 65.5 (60 – 70) | | 0.459 | 66 (55 – 75) | | 67.5 (56 – 76.8) | | 60.5 (51.8 – 72.3) | | 0.139 |
| **BMI** (median. IQR) | 25.8 (23.2 - 28.4) | | 25.7 (23.4 – 28.4) | | 25.9 (23.2 – 28.6) | | 0.808 |  |  |  | |  |  |  |
| **Tumor stage** |  |  |  |  |  |  | 0.503 |  |  |  |  |  |  | 0.180 |
| I | 40 | (27.4) | 33 | (26.6) | 7 | (31.8) |  | 28 | (20) | 21 | (19.3) | 7 | (31.8) |  |
| II | 63 | (43.2) | 56 | (45.2) | 7 | (31.8) |  | 48 | (34.3) | 42 | (38.5) | 6 | (27.3) |  |
| III | 43 | (29.4) | 35 | (28.2) | 8 | (36.4) |  | 35 | (25) | 27 | (24.8) | 8 | (36.4) |  |
| IV | 0 | 0 |  |  |  |  |  | 20 | (14.3) | 19 | (17.4) | 1 | (4.5) |  |
| Missing |  |  |  |  |  |  |  | 9 | (6.4) |  |  |  |  |  |
| **pT-stage** |  |  |  |  |  |  | 0.742 |  |  |  |  |  |  | 0.118 |
| Tis | 0 | 0 | 0 | 0 | 0 | (0) |  | 1 | (0.7) | 0 | 0 | 1 | (4.5) |  |
| 1 | 0 | 0 | 0 | 0 | 0 | (0) |  | 5 | (3.6) | 4 | (3.4) | 1 | (4.5) |  |
| 2 | 46 | (31.5) | 38 | (30.6) | 8 | (36.4) |  | 27 | (19.3) | 21 | (18.1) | 6 | (27.4) |  |
| 3 | 98 | (67.1) | 84 | (67.7) | 14 | (63.6) |  | 89 | (63.6) | 76 | (65.6) | 13 | (59.1) |  |
| 4 | 2 | (1.4) | 2 | (1.6) | 0 | 0 |  | 16 | (11.4) | 15 | (12.9) | 1 | (4.5) |  |
| Missing |  |  |  |  |  |  |  | 2 | (1.4) |  |  |  |  |  |
| **pN-stage** |  |  |  |  |  |  | 0.735 |  |  |  |  |  |  | 0.363 |
| 0 | 104 | (71.2) | 90 | (72.6) | 14 | (63.6) |  | 83 | (59.3) | 70 | (60.3) | 13 | (59.1) |  |
| 1 | 28 | (19.2) | 22 | (17.7) | 6 | (27.3) |  | 38 | (27.2) | 30 | (25.9) | 8 | (36.4) |  |
| 2 | 14 | (9.6) | 12 | (9.7) | 2 | (9.1) |  | 17 | (12.1) | 16 | (13.8) | 1 | (4.5) |  |
| Missing |  |  |  |  |  |  |  | 2 | (1.4) |  |  |  |  |  |
| **Tumor differentiation** |  |  |  |  |  |  | 0.000 |  |  |  |  |  |  |  |
| Good | 14 | (9.6) | 14 | (11.3) | 0 | (0.0) |  |  |  |  |  |  |  |  |
| Moderate | 123 | (84.2) | 107 | (86.3) | 16 | (72.7) |  |  |  |  |  |  |  |  |
| Poor | 8 | (5.5) | 3 | (2.4) | 5 | (22.8) |  |  |  |  |  |  |  |  |
| Unknown/Other | 1 | (0.7) | 0 | (0) | 1 | (4.5) |  |  |  |  |  |  |  |  |
| **Tumor location** |  |  |  |  |  |  | 0.230 |  |  |  |  |  |  | 0.008 |
| Right | 54 | (37) | 43 | (34.7) | 11 | (50) |  | 71 | (50.7) | 54 | (45.7) | 17 | (77.3) |  |
| Left | 92 | (63) | 81 | (65.3) | 11 | (50) |  | 61 | (43.6) | 57 | (48.3) | 4 | (18.2) |  |
| Missing |  |  |  |  |  |  |  | 8 | (5.7) | 7 | (6) | 1 | (0.05) |  |
| **Rectum/Colon** |  |  |  |  |  |  |  |  |  |  |  |  |  | 0.751 |
| Colon | 146 | (100) |  |  |  |  |  | 137 | (97.9) | 115 | (97.5) | 22 | (100) |  |
| Rectum | 0 | 0 |  |  |  |  |  | 1 | (0.7) | 1 | (0.8) | 0 | 0 |  |
| Missing |  |  |  |  |  |  |  | 2 | (1.4) | 2 | (1.7) | 0 | 0 |  |
| **Adjuvant therapy** |  |  |  |  |  |  | 0.446 |  |  |  |  |  |  |  |
| No | 104 | (71.2) | 90 | (72.6) | 14 | (63.6) |  |  |  |  |  |  |  |  |
| Yes | 42 | (28.8) | 34 | (27.4) | 8 | (36.4) |  |  |  |  |  |  |  |  |
